# Alectinib shows promise for the treatment of refractory ALK-positive large B-cell lymphoma: a case report

**DOI:** 10.3389/fonc.2026.1844016

**Published:** 2026-06-17

**Authors:** Atheer Alsulami, Roula Almufti, Saleem Eldadah, Yahya Al-Ghamdi

**Affiliations:** 1 Department of Adult Medical Oncology, Princess Noorah Oncology Center, King Abdulaziz Medical City, Jeddah, Saudi Arabia; 2 Department of Adult Medical Oncology, King Faisal Specialist Hospital and Research Center, Jeddah, Saudi Arabia; 3 Department of Adult Hematology and Stem Cell Transplantation, Princess Noorah Oncology Center, King Abdulaziz Medical City, Jeddah, Saudi Arabia; 4 Department of Pathology, Umm Al-Qura University, Makkah, Saudi Arabia

**Keywords:** alectinib, ALK inhibitors, ALK-positive large B-cell lymphoma (ALK+ LBCL), case report, refractory

## Abstract

**Introduction:**

Anaplastic lymphoma kinase-positive large B-cell lymphoma (ALK+ LBCL) is rare, accounts for <1% of DLBCL, and is characterized by ALK rearrangement. ALK+ LBCL is a very aggressive disease with a poor prognosis, a high risk of relapse and a poor response to standard chemotherapy treatment, with a median overall survival of approximately one year in advanced-stage disease. The activity of ALK inhibitors in the treatment of relapsed/refractory ALK+ LBCL is not well studied. Here, we report our experience with the use of alectinib in refractory ALK+ LBCL.

**Case presentation:**

A previously healthy 14-year-old Saudi Arabian girl presented with progressive chest, loin, and iliac crest pain over two months, without B symptoms. Imaging revealed a left supradiaphragmatic soft tissue mass with supra and infradiaphragmatic nodal masses, pancreatic mass and right iliac bone aggressive lesion. Core biopsy of a left pararenal mass confirmed ALK-positive large B-cell lymphoma (ALK+ LBCL). Immunohistochemistry showed positivity for CD138, MUM1, CD79a (subset), CD4, EMA, CD45, CD10 (weak), BCL6, c-MYC, BCL2, and cytoplasmic ALK1. CD20 and PAX5 were negative. ALK rearrangement was confirmed by FISH; the bone marrow was not involved. Initial treatment with CHOEP failed, followed by progression on salvage ESHAP and IGEV chemotherapy. Radiation was given for bulky disease. Crizotinib was initiated with partial response but discontinued due to hepatotoxicity and disease progression. Alectinib was then started, resulting in a complete metabolic response (CMR). This was consolidated with an allogeneic stem cell transplant from a matched sibling donor, leading to a sustained CMR for 4 years with good tolerance.

**Conclusion:**

The use of next-generation ALK inhibitors in this case demonstrated excellent tolerability and induced sustained responses. This report suggests that alectinib may be an effective bridging therapy for refractory ALK+ LBCL to enable allogeneic SCT in selected patients. Further prospective studies are warranted to explore and optimize therapeutic strategies for this rare disease.

## Introduction

The World Health Organization (WHO) categorizes almost 100 distinct types of lymphoid malignancies (1). The vast variety of lymphoid neoplasms mirrors the complexity of the immune system, encompassing both its stages of differentiation and its functional range (2).ALK+ LBCL was initially identified in 1997 and described as an uncommon, aggressive subtype of diffuse large B-cell lymphoma (DLBCL), accounting for less than 1% of all DLBCL cases, and since then, approximately 100 cases have been documented in the medical literature (3, 4). In the 2008–2022 World Health Organization Classification of Lymphoid Neoplasms (4th–5th edition), ALK+ LBCL is recognized as a distinct type of mature B-cell neoplasm (5).

ALK+ LBCL affects both pediatric and adult patients, primarily presenting as advanced disease with involvement of the lymph nodes and/or extranodal sites (3). It is more prevalent in men, with a male-to-female ratio of 5:1, and occurs across a broad age range of 14–85 years (4).

Anaplastic lymphoma kinase (ALK) expression plays a significant role in various hematopoietic and non-hematopoietic neoplasms, first identified in anaplastic large-cell lymphoma (ALCL) through the t(2;5)(p23;q35) translocation, which results in the formation of the nucleophosmin (NPM)-ALK fusion protein (6, 7). Other neoplasms that exhibit ALK expression due to ALK gene rearrangements include ALK+ histiocytosis, anaplastic large-cell lymphoma (ALCL), inflammatory myofibroblastic tumors, and non-small cell lung cancer (8). ALK-positive large B-cell lymphoma (ALK+ LBCL) is an aggressive subtype characterized by the absence of common B-cell markers such as CD20, CD79a, and PAX5, as well as T-cell markers like CD2 and CD3, and CD30. Instead, ALK+ LBCL tumor cells frequently express markers of terminal B-cell differentiation and plasma cell phenotype, including CD38, CD138, MUM1, and VS38c, with epithelial membrane antigen (EMA) expressed in 97% of cases (9, 10). Diagnosing ALK+ LBCL can be particularly challenging due to its rarity, limited awareness, and considerable morphologic and immunophenotypic resemblance to other hematopoietic and non-hematopoietic neoplasms (3), such as plasmablastic lymphoma (PBL), which are distinguished primarily by ALK expression and viral associations. ALK+ LBCL is defined by strong ALK protein expression, often with a granular cytoplasmic staining pattern, and often shows a sinusoidal growth pattern within lymph nodes. In contrast, PBL is predominantly an extranodal lymphoma with a diffuse growth pattern. Furthermore, PBL is strongly associated with EBV/EBER positivity and underlying immunodeficiency, such as HIV infection, whereas ALK+ LBCL is EBV negative and occurs independently of immune status. Genetically, ALK+ LBCL is driven by *ALK* gene rearrangements, whereas PBL more often harbors *MYC* rearrangements and lacks *ALK* rearrangement (11). Recognizing the distinctive molecular and immunophenotypic features of ALK+ LBCL is essential for timely and accurate diagnosis.

ALK-positive large B-cell lymphoma (ALK+ LBCL) exhibits aggressive clinical behavior, characterized by a high relapse rate and poor response to standard chemotherapy, with a median overall survival of approximately one year in patients with advanced-stage disease (4, 10). The dismal prognosis is largely attributed to its resistance to conventional chemoimmunotherapy and the absence of effective targeted therapeutic options (3). Hence, exploring the potential applications of novel agents, particularly ALK inhibitors, is of critical importance.

ALK inhibitors exert their effect by binding to the ATP-binding site of the ALK tyrosine kinase domain, thereby preventing its activation and subsequent signaling (12).

Crizotinib, a first-generation oral tyrosine kinase inhibitor targeting ALK, MET, and ROS1, has demonstrated efficacy in ALK-rearranged malignancies such as ALCL (13, 14).

The response to crizotinib is more durable in ALK+ ALCL, which typically harbors NPM-ALK fusion in contrast to the CLTC-ALK fusion commonly observed in ALK+ LBCL (15).

Alectinib and lorlatinib are next-generation ALK inhibitors that are more potent than crizotinib and are effective in crizotinib-refractory ALK-positive cancers (16, 17).

The use of next-generation ALK inhibitors is also favored over use of first-generation inhibitors in the NCCN guidelines® (18).

Although ALK inhibitors have demonstrated clinical benefit in other ALK-rearranged malignancies, such as ALK-positive non-small cell lung cancer and ALCL (19), their role in ALK+ LBCL is not well defined, with anecdotal reports of crizotinib use along with cytotoxic chemotherapy showing limited success (13, 14).

In this report, we describe a case of refractory ALK+ LBCL that responded dramatically to alectinib after failure to respond to multiple chemotherapy regimens. This case adds to the growing evidence supporting the potential role of targeted ALK inhibition for the treatment of this rare disease entity.

## Case presentation

We present the case of a 14-year-old Saudi Arabian girl with no significant past medical history.

Her symptoms started with vague chest discomfort, left loin pain and right iliac crest pain, with no B symptoms. These symptoms progressed over a period of 2 months.

Her CT scans revealed a left supradiaphragmatic soft tissue mass touching the left paracardial surface/cardiac apex, causing invasion of the left side of the pericardium with surrounding pleural and pericardial effusion. She had mildly enlarged epicardial, internal mammary, hilar, sub-carinal and para-aortic lymph nodes along with a pancreatic mass and a destructive lesion in the right iliac bone (stage IV - Ann Arbor classification).

The patient subsequently underwent left pararenal mass core needle biopsy, which revealed a diffuse proliferation of atypical large lymphoid cells that had a plasmablastic morphology and exhibited eccentric nuclei, vesicular chromatin, prominent nucleoli, and abundant amphophilic cytoplasm. Large and atypical HRS-like cells were observed. To further characterize the neoplastic population, immunohistochemical and molecular analyses were performed. By immunostaining, the tumor cells were positive for CD138 (strong), MUM1 (variable), CD79a (variable, subset), CD4, EMA, CD45, CD10 (weak), BCL6, c-MYC, BCL2, and ALK1 (cytoplasmic, granular pattern), and negative for CD20, PAX5, CD3, CD30, CD38, HHV8, and EBV-ISH (EBER). FISH for ALK1 was positive for rearrangement (but the gene partner was not determined) and the bone marrow was not involved. These findings are diagnostic of ALK+ LBCL (**Figure 1**).

**Figure 1:**
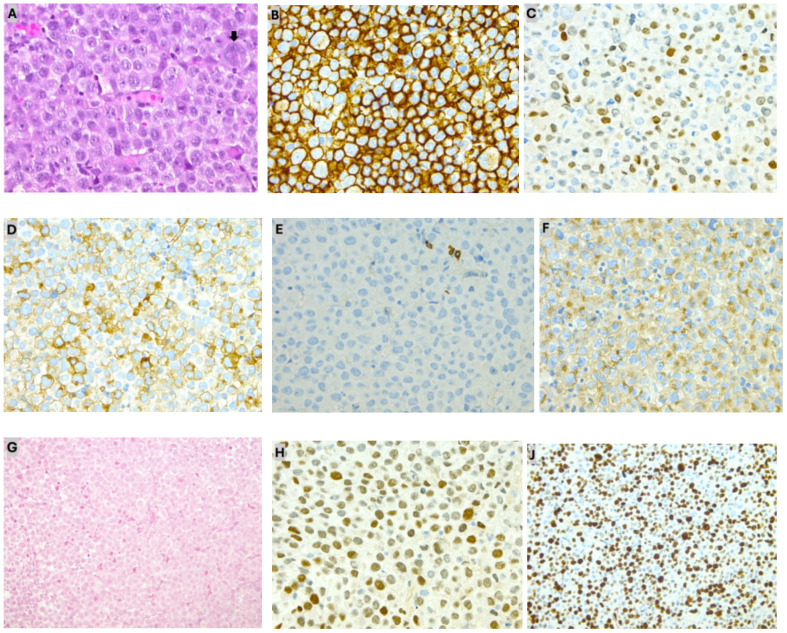
**(A)** The tumor cells had a plasmablastic morphology with eccentric nuclei, vesicular chromatin, prominent nucleoli, abundant amphophilic cytoplasm, and large atypical HRS-like cells (arrowhead). The tumor cells were positive for CD138 **(B)**, MUM1 **(C)**, CD79a **(D)**, BCL6 **(H)**, and ALK1 by IHC **(F)** and negative for EBV-encoded RNA (EBER) **(G)** and CD20 **(E)**. The Ki-67 proliferation index was approximately 80% (200×), as shown in **(J)**.

the CHOEP protocol was started, but unfortunately, the patient progressed after 2 cycles, based on a PET/CT scan findings demonstrating new progressive lymph nodes in the mediastinum and left supraclavicular region. A left Supraclavicular lymph node was biopsied, confirming the same diagnosis.

The patient subsequently started the salvage ESHAP protocol and received 2 cycles. Follow-up CT scans revealed a significant increase in the size of the cervical lymph nodes, mainly in both supraclavicular areas, causing local infiltration to the left sternocleidomastoid muscle and compression along with thrombosis of the left internal jugular vein and several newly observed prominent retroperitoneal lymph nodes.

Afterward, the patient underwent radiotherapy targeting the left supraclavicular and cervical masses and was started on a second salvage treatment cycle with an IGEV protocol. However, unfortunately, she progressed after 2 cycles based on CT scan images showing further increases in the size of the intra-abdominal lymph nodes. After that, the patient commenced crizotinib treatment (250 mg twice daily) with objective improvement in chest, left loin, and right iliac crest pain.

However, she had grade II-III hepatotoxicity; crizotinib was paused for 2 weeks then resumed at 200 mg twice daily after normalization of AST/ALT levels.

Despite an initial partial response (Deauville 4) after 3 months, disease progression was observed after 6 months of treatment based on PET/CT scans (Deauville score of 5b).

After a multidisciplinary meeting, the patient was started on alectinib at 450 mg twice daily. She had excellent tolerance and a remarkable clinical response. HLA tissue typing revealed a fully matched brother, and after discussion at the BMT tumor board, we decided to consolidate her response with allogenic stem cell transplantation (SCT). She underwent oocyte cryopreservation prior to MSD-MAC allogeneic SCT and conditioning with cyclophosphamide/total body irradiation (GVHD PPx: cyclosporine and methotrexate; ABO-Mismatched O+/B+; and CMV: both nonreactive). D100 BM showed complete chimerism, and the patient developed mild skin graft-versus-host disease (GVHD) manifested as a maculopapular rash involving both hands that responded well to topical steroids. Alectinib was resumed 4 months post-HSCT and continued for 4 years as maintenance.

Radiologically, the patient showed no definite residual avid lymphadenopathy (complete metabolic response) on PET/CT scan, which was repeated 2, 10, and 17 months later (**Figures 2A, B**). The patient received a COVID-19 vaccination as per Saudi ministry of health regulations at that time.

**Figure 2:**
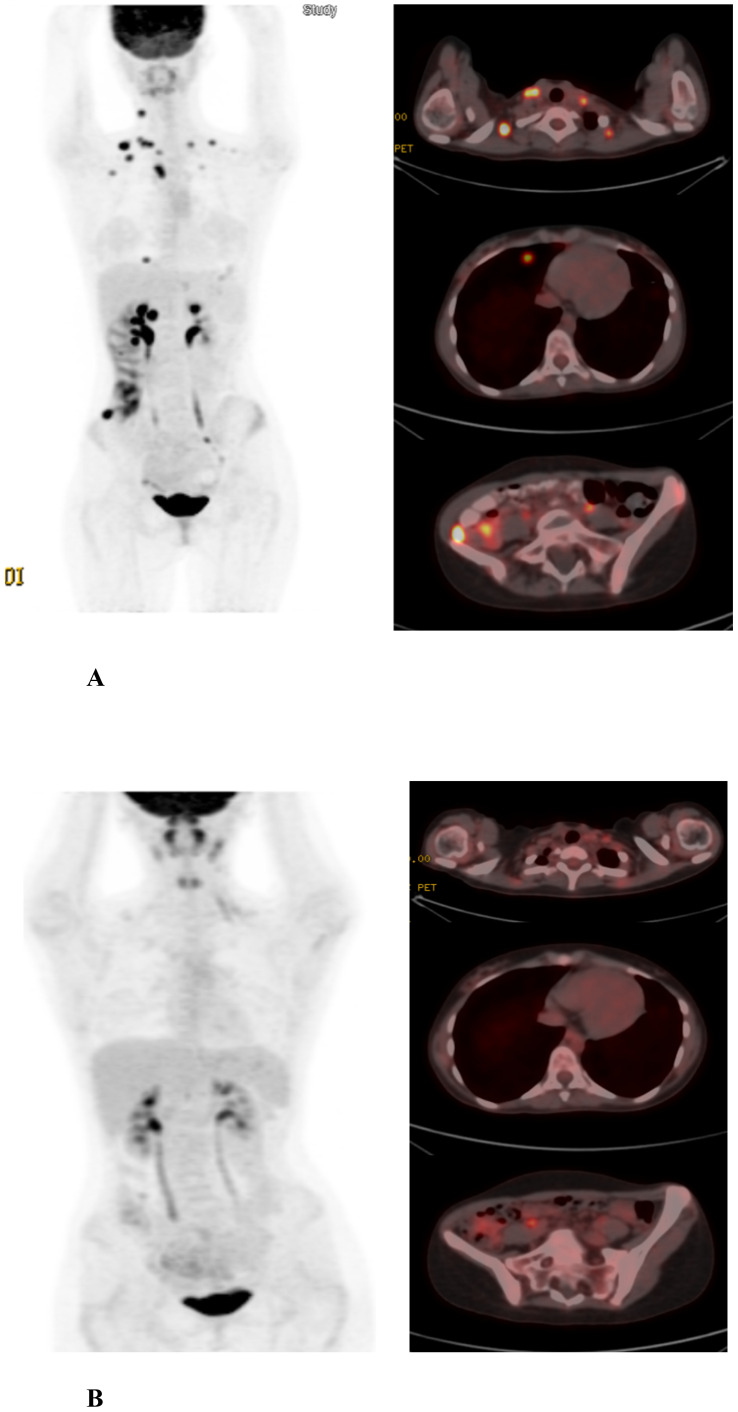
**(A)** PET/CT images obtained while the patient was on crizotinib (prealectinib), showing markedly intense, metabolically active bilateral lower posterior cervical and bilateral supraclavicular lymph nodes and moderate uptake in a new 1.2 cm right middle pulmonary nodule, as well as intense uptake in right iliac fossa lesions: Deauville score of 5b. **(B)** PET/CT images obtained after 6 months of alectinib treatment didn't show any residual avid lymphadenopathy in keeping with complete metabolic response (Deauville score of 3).

## Discussion

This case highlights the potential of alectinib to induce a durable remission in ALK-positive LBCL refractory to multiple lines of chemotherapy, with a sustained response following allogeneic stem cell transplantation. Given the rarity and limited treatment options for this aggressive lymphoma, our findings contribute to the evolving therapeutic landscape.

ALK+ LBCL is a type of rare CD20-negative invasive non-Hodgkin lymphoma (20). The CLTC-ALK fusion protein drives constitutive ALK activity, on which ALK+ LBCL appears to be dependent (21).

The clinical features of patients with ALK+ LBCL closely resemble those of patients with DLBCL, who typically present with painless lymphadenopathy at onset, rapid disease progression, widespread nodal involvement, and frequent extranodal extension. Common sites of extranodal involvement include soft tissues, the nasopharynx, the tonsils, the thoracic wall, the mediastinum, the pulmonary hilum, the liver, and the spleen, whereas bone marrow and cerebrospinal fluid involvement are uncommon (3, 22). There is no established standard treatment for ALK+ LBCL: most patients receive B-cell lymphoma-based chemotherapy such as CHOEP or DA-EPOCH. CHOP may also be considered for patients with early disease as part of combined modality therapy, although it is associated with sub-optimal outcomes (18). According to the NCCN guidelines®, Mini-CHOP, HyperCVAD and CODOX-M alternating with IVAC are also possible treatment options that should be considered in a selected group of patients (18).

In relapsed/refractory settings, participation in a clinical trial is recommended when available. For transplant eligible patients with chemosensitive disease, second-line platinum-based chemotherapy followed by highdose therapy (HDT) and autologous stem cell rescue (ASCR) should be considered (18).

Pan et al. (3) reported 26 cases of ALK+ LBCL and reviewed an additional 108 from the literature; most patients were treated with CHOP, CHOEP, EPOCH, or CVAD, with a subset also receiving localized radiotherapy and hematopoietic stem cell transplantation. Despite these approaches, ALK+ LBCL demonstrated markedly poor clinical outcomes compared with typical DLBCL treated with CHOP or CHOP-like regimens. Among 91 patients with available survival data, 49% (45/91) died during a median follow-up of 1.8 years. The 5-year overall survival (OS) rate was 34%, with a median survival of 1.83 years. Patients with stage I-II disease have better 5-year overall survival (OS) than those with stage III-IV disease (66% and 8%, respectively).

Similarly, Laurent et al. (4) reported 38 patients treated with CHOP-based regimens, demonstrating a median overall survival of 12.2 months in patients with advanced-stage disease.

On the basis of these findings, we conclude that only a minority of patients achieve long-lasting responses following first-line chemotherapy. Therefore, a shift toward more targeted therapy such as ALK inhibitors should be considered.

The anaplastic lymphoma kinase (ALK) gene plays a key oncogenic role in several malignancies through constitutive activation resulting from gene rearrangements such as NPM-ALK and TPM3-ALK. Preclinical studies using mouse models suggest that overexpression of these fusion oncogenes may disrupt early B-cell differentiation, contributing to the development of ALK-positive B-cell lymphoproliferative disorders; notably, these effects can be reversed with ALK inhibitor therapy (23). Supporting this, preclinical *in vitro* studies of ALK+ large B-cell lymphoma (LBCL) have shown that ALK inhibition can suppress tumor proliferation and induce regression (21, 29).

Takiar et al. (25) reported 1 patient achieved a CR with crizotinib for 6 years, and the other patient achieved an initial response followed by PD after 2 months of crizotinib use.

Furthermore, Soumerai et al. (24) described 3/3 patients who were refractory to prior crizotinib treatment. They hypothesized that in the absence of ALK resistance mutations or gene amplifications, crizotinib resistance in ALK+LBCL can be explained by upregulation of bypass signaling pathways possibly driven by the tumor microenvironment or from P-glycoprotein-mediated drug efflux, with more potent ALK inhibitors potentially overcoming these mechanisms.

Xia et al. (28) reported an acquired resistance to crizotinib identified by next-generation sequencing in a patient with ALK+LBCL who developed progressive disease approximately 5.5 months after treatment with a combination of crizotinib and chemotherapy. Next-generation sequencing after progression revealed that CDK14-ALK abundance decreased from 20.16% to 6.08%, CLTC-ALK increased from 2.71% to 6.34%, MFHAS1 increased markedly from 0.32% to 31.01%, and the results for the MTOR gene turned negative. The identification of a CDK14-ALK fusion gene in this case was novel. The authors suggested that the CDK14-ALK fusion gene may be more sensitive to crizotinib than the CLTC-ALK fusion gene is. MFHAS1 mutations and/or CLTC-ALK copy number amplification may contribute to crizotinib resistance. In addition, MTOR is implicated in the antitumor activity of ALK inhibitors, and alectinib is more efficacious for these alterations.

Atallah et al. (15) conducted the largest multi-institutional retrospective study of patients with ALK+ LBCL to date. They analyzed 39 patients with ALK+ LBCL from 2002 to 2024; 12/39 patients received ALK inhibitors, with alectinib producing a more sustained response than crizotinib did.

Similarly, Soumerai et al. (24) developed the first patient-derived xenograft (PDX) model of ALK+ LBCL and showed that a highly potent ALK inhibitor led to complete suppression of ALK phosphorylation and substantial tumor reduction. In accordance with these encouraging findings, alectinib has been used to treat 4 patients with relapsed/refractory ALK+ LBCL who had not achieved a durable complete response with prior therapies. Two out of the four patients maintained a CR with alectinib (600 mg twice daily) and subsequently received a myeloablative stem cell transplant. One of the two patients developed progressive disease and was subsequently treated with lorlatinib, achieving a complete response as indicated by PET/CT scan, demonstrating that transitioning between high-potency ALK inhibitors might also be beneficial.

Further support for the efficacy of alectinib in ALK+ LBCL comes from a recent report describing a 53-year-old male diagnosed with ALK-positive large B-cell lymphoma. The patient was treated with alectinib combined with vincristine, rituximab, cyclophosphamide, and doxorubicin (VRCD). Brentuximab vedotin was added in the fourth cycle to enhance treatment efficacy. After four cycles, PET-CT and bone marrow evaluation confirmed a complete response, enabling successful stem cell collection and subsequent autologous hematopoietic stem cell transplantation. The patient remains in favorable condition while currently receiving maintenance therapy with alectinib and lenalidomide. This finding suggests that, in the context of precision oncology, the integration of targeted agents with conventional chemotherapy may enhance therapeutic efficacy and improve the prognosis of rare lymphoma subtypes, including ALK-positive large B-cell lymphoma (ALK+ LBCL) (27) (**Table 1**).

**Table 1:** Clinicopathologic and outcome comparison of our case with published cases.

Case	Age at diagnosis (years)	Immunophenotyping	Treatment	Complication and side effects	Response
Our case	14 years	Positive for CD45, CD79a (weak), CD4, EMA, and ALK-1 (cytoplasmic, granular), and CD138, MUM1 and Ki-67 (up to 80%) and negative for CD20, PAX-5, CD3, CD5, CD43, CD3Q, CD15, EBV-EBER and HHV8.	Chemotherapy, (CHOEP, ESHAP, IGEV) crizotinib alectinib	Transient rise in liver enzymes with crizotinib managed by holding the drug shorty with conservative management.	achieved CR with alectinib for 4 years.
Soumerai, et al. (24)	25–50 years (4 patients)	Negative for CD20, CD19, PAX5, CD3, CD2 and CD5.	Chemotherapy, (CHOP, EPOCH, DHAP, ICE, lenalidomide, bendamustine, Gem-Ox, and Elotuzumab), crizotinib alectinib	No reported toxicity.	2/4 patients achieved CR with alectinib and 2/4 patients developed PD; one subsequently received lorlatinib 100 mg PO daily off-label and achieved CR clinically and radiologically
Takiar, et al. (25)	23 years and 46 years (2 patients)	Patient A; Positive for ALK, EMA, MUM1, CD45 (weak), CD79a (weak), CD138 (subset). And negative for CD3, CD20, CD30 CD43, HHV8, Melan-A, OCT4, Pan-keratin- and S100 Patient B; Positive for ALK, CD45, CD138, and PAX5 (weak), CD4 (weak), CD30 (weak, subset), IgA heavy chain, and kappa light chain (weak). In addition, negative for CD20, CD3, CD5, CD8, S100, MCK and lambda light chain.	Chemotherapy (CHOP), Radiation, crizotinib Chemotherapy (CHOP), radiation, crizotinib, alectinib lorlatinib	Incidentally found Subsegmental Pulmonary embolism treated with Rivaroxaban Transient rise in liver enzymes seen with crizotinib (AST 151 U/L and ALT 323 U/L) resolved with hydration and conservative management, COVID-19 and Incidental finding of subsegmental Pulmonary embolism treated with Apixaban.	Patient A: achieved CR with crizotinib for 6 years Patient B: achieved PR with alectinib for 6 months but subsequently developed PD; switched to lorlatinib and achieved CR for 6 months
Li et al (26)	21 years	Positive for CD138, CD4, MUM1, ALK, CD30 (weak), CD38 (weak), CD43 (weak), and CD57 (weak); negative for CD20, CD15, CD79a, CD3, CD7, and S100	Chemotherapy (CHOP, ICE, Gem-Ox + Dexamethasone + crizotinib)	No reported toxicity.	Short-lived response to Gem-Ox + Dexamethasone + crizotinib followed by rapid PD and death.
Wang et al (27)	53 years	Positive for ALK (90%+, cytoplasm), CD4, CD138, MUM1, Ki67 (80%), LCA (weak), EMA (partially +), PAX5 (weak); negative for CD3, CD5, CD20, CD79a, CK7, S100, CD43, CD30 (25%+), CD38, CD163, and EBER	Chemotherapy (VRCD + alectinib + BV added with cycle 4) + HSCT followed by maintenance alectinib and lenalidomide	Bradycardia without syncope or palpitation; no intervention was needed.	Achieved CR for 2 years.
Xia et al (28)	15 years	Positive for ALK, CD138, EMA, MUM-1, OCT2, Bob.1, and CD38 (partial); negative for CD30, CD20, PAX5, and CD5. Ki-67 proliferation index was 60%.	CDOP + crizotinib, CHOPE + crizotinib, DHAP, Hyper-CVAD ×1 + alectinib followed by maintenance alectinib	No reported toxicity.	Achieved PR for 28 months.
Atallah et al (15)	>18 years of age (12 patients)	Not reported	Anthracycline-based chemotherapy (CHOP, CHOEP, min-CHOP, R-CHOP, MR-CHOP, BVCHP, CVP), HyperCVAD, CODOX-M/IVAC, DA/R-EPOCH, crizotinib, alectinib	Rash with alectinib; patient switched to crizotinib.	5/8 patients developed PD to crizotinib, while 1/8 had short-lived remission for less than 1 month and 1/8 achieved CR with crizotinib + pembrolizumab for 1–2 months followed by allo-SCT. SD was observed in 1/8 for 4 months. With alectinib, 1/4 achieved CR for 2 years, 1/4 achieved CR after allo-SCT, and 1/4 had PR then switched to lorlatinib upon progression and achieved another PR.

Similar to our patient, Atallah et al. (15) described a patient who achieved a partial response with Alectinib and converted to complete remission following allo-SCT. The patient resumed alectinib on post-transplant day 60 and continued maintenance therapy for 2 years, remaining in sustained CR at the last follow-up, 4.4 years later. This finding highlights the importance of allogenic SCT following CR to ALK inhibitors.

Consideration of CD-19 directed CAR T-cell therapy is also important, although in our case, CD19 was not done because it was unavailable (18).

This case report is limited by its single-patient design, which restricts generalizability. The relative contribution of alectinib to the sustained remission remains unclear. These limitations underscore the need for larger studies integrating clinical and molecular data to guide management of ALK+ LBCL.

## Patient perspective

The patient was very mature for her age, and she understood the disease by reading about it. She had a very positive attitude and was well supported by her family. The family had very strong religious beliefs, and even when the patient progressed on the third line of treatment, she remained at peace and still deeply believed that God would cure her.

## Conclusion

On the basis of these promising findings concerning the treatment of refractory ALK+ LBCL, further prospective studies are needed as a potential bridging strategy to allogeneic SCT in selected patients.

## Data Availability

The raw data supporting the conclusions of this article will be made available by the authors, without undue reservation.

## Glossary

ALK+ LBCL: Anaplastic lymphoma kinase-positive large B-cell lymphoma
DLBCL: Diffuse Large B-cell lymphoma
ALK: Anaplastic lymphoma kinase
CD38: Cluster of Differentiation 38
MUM1: Multiple Myeloma Oncogene 1
CD79a: Cluster of Differentiation 79a
CD4: Cluster of Differentiation 4
EMA: Epithelial Membrane Antigen
CD45: Cluster of Differentiation 45
CD10: Cluster of Differentiation 10
CD19: Cluster of Differentiation 19
CD20: Cluster of Differentiation 20
BCL6: B-cell lymphoma 6 protein
BCL2: B-cell lymphoma 2 protein
c-MYC: Cellular Myelocytomatosis oncogene
PAX5: Paired Box 5
HHV8: Human Herpesvirus 8
HIV: Human immunodeficiency virus
EBV-ISH (EBER): Epstein-Barr Virus in Situ Hybridization for Epstein-Barr encoded RNA
FISH: Fluorescence in Situ Hybridization
CHOEP: Chemotherapy protocol that contains (Cyclophosphamide, Doxorubicin, Vincristine, Etoposide and Prednisone)
ESHAP: Chemotherapy protocol that contains (Etoposide, Methylprednisolone, Cytarabine and Cisplatin)
IGEV: Chemotherapy protocol that contains (Ifosfamide, Gemcitabine, Vinorelbine, Prednisone or sometimes Vinblastine (regimens can vary)
CMR: Complete Metabolic Response
WHO: World Health Organization
ALCL: Anaplastic large-cell lymphoma
ALKi: Anaplastic lymphoma kinase Inhibitors
CT: Computed scan
PET scan: Positron Emission Tomography scan
FDG: Fluorodeoxyglucose
AST/ALT: Aspartate Aminotransferase/Alanine Aminotransferase
LFTs: Liver function tests
HLA: Human leukocyte antigen
BMT: Bone marrow transplant
SCT: stem cell transplantation
HSCT: Hematopoietic stem cell transplantation
MSD-MAC: Matched Sibling Donor-Myeloablative Conditioning
GVHD: graft-versus-host disease
PPx: Prophylaxis
CMV: Cytomegalovirus
NGS: Next-generation-sequencing
D100BM: Day 100 bone marrow
CLTC-ALK fusion protein: Clathrin Heavy Chain- Anaplastic Lymphoma Kinase
NPM-ALK: Nucleophosmin-Anaplastic Lymphoma Kinase
TPM3-ALK: Tropomyosin 3-Anaplastic Lymphoma Kinase
MET: *Mesenchymal-Epithelial Transition factor*
ROS1: *ROS Proto-Oncogene 1*
PDX: patient-derived xenograft
CR: Complete response
PR: Partial response
EPOCH: Chemotherapy protocol that contains (etoposide, prednisone, vincristine, cyclophosphamide and hydroxydaunorubicin)
DA-EPOCH: Dose-adjusted EPOCH
DHAP: Chemotherapy protocol that contains (Dexamethasone, High-dose Ara-C and Cisplatin)
ICE: Chemotherapy protocol that contains (Ifosfamide, Carboplatin and Etoposide)
Gem-Ox: Chemotherapy protocol that contains (Gemcitabine and Oxaliplatin)
VRCD: Chemotherapy protocol that contains (Vincristine, Rituximab, Cyclophosphamide and Doxorubicin)
NCCN: National Comprehensive Cancer Network
Hyper-CVAD: Hyperfractionated chemotherapy that contains Cyclophosphamide, Vincristine, Doxorubicin and Dexamethasone
CODOX-M alternating with IVAC: Chemotherapy protocol that contains CODOX-M
Cyclophosphamide: Vincristine, Doxorubicin and high dose Methotrexate. IVAC
Ifosfamide: Etoposide and Cytarabine
BV-CHP: Chemotherapy protocol that contains Brentuximab vedotin, Cyclophosphamide, Doxorubicin and Prednisone
MR-CHOP: Chemotherapy protocol that contains Methotrexate, Rituximab, Cyclophosphamide, Doxorubicin, Vincristine and Prednisone
R-CHOP: Chemotherapy protocol that contains Rituximab, Cyclophosphamide, Doxorubicin, Vincristine and Prednisone
CDK14-ALK: Cyclin Dependent Kinase 14- Anaplastic Lymphoma Kinase
mTOR: mechanistic Target of Rapamycin
CAR-T: Chimeric Antigen Receptor T-cell therapy.
